# The correlation between diverticulosis and redundant colon

**DOI:** 10.1007/s00384-017-2894-5

**Published:** 2017-09-20

**Authors:** Tahleesa Cuda, Ronny Gunnarsson, Alan de Costa

**Affiliations:** 1 Cairns Clinical School, College of Medicine and Dentistry, James Cook University, Cairns, QLD 4870 Australia; 2 Department of Surgery, Cairns Private Hospital, Cairns, QLD Australia; 3 Research and Development Unit, Primary Health Care and Dental Care, Southern Älvsborg County, Region Västra Götaland, Vänersborg, Sweden; 4 Department of Public Health and Community Medicine, Institute of Medicine, The Sahlgrenska Academy, University of Gothenburg, Gothenburg, Sweden

**Keywords:** Diverticulosis, Redundant colon, Acquired megacolon, Idiopathic megacolon, Colonoscopy

## Abstract

**Background:**

Diverticulosis and redundant colon are colonic conditions for which underlying pathophysiology, management and prevention are poorly understood. Historical papers suggest an inverse relationship between these two conditions. However, no further attempt has been made to validate this relationship. This study set out to assess the correlation between diverticulosis and colonic redundancy.

**Methods:**

Redundant colon, diverticulosis and patient demographics were recorded during colonoscopy. Multivariate binary logistic regression was performed with redundant colon as the dependent variable and age, gender and diverticulosis as independent variables. Nagelkerke *R* ^2^ and a receiver operator curve were calculated to assess goodness of fit and internally validate the multivariate model.

**Results:**

Redundant colon and diverticulosis were diagnosed in 31 and 113 patients, respectively. The probability of redundant colon was increased by female gender odds ratio (OR) 8.4 (95% CI 2.7–26, *p* = 0.00020) and increasing age OR 1.7 (95% CI 1.1–2.6, *p* = 0.017). Paradoxically, diverticulosis strongly reduced the probability of redundant colon with OR of 0.12 (95% CI 0.42–0.32, *p* = 0.000039). The Nagelkerke *R* ^2^ for the multivariate model was 0.29 and the area under the curve at ROC analysis was 0.81 (95% CI 0.73–0.90 *p*–value 3.1 × 10^−8^).

**Conclusions:**

This study found an inverse correlation between redundant colon and diverticulosis, supporting the historical suggestion that the two conditions rarely occur concurrently. The underlying principle for this relationship remains to be found. However, it may contribute to the understanding of the aetiology and pathophysiology of these colonic conditions.

## Introduction

Diverticulosis and redundant colon are colonic conditions posing significant morbidity and mortality risks for those affected [1, 2]. Despite this, the pathophysiological mechanisms, management and prevention of these conditions are not well understood [3, 4].

### Diverticulosis

Diverticulosis is the most common pathological condition of the colon, affecting 10% of individuals under the age of 40 years and 50–70% of those greater than 80 years of age. Suggestions of altered connective tissue and enteric neuropathy exist, although the exact pathological mechanisms remain unknown [5–9].

Diverticulosis pertains to the presence of the colonic diverticula, regardless of clinical significance. Symptomatic uncomplicated diverticular disease has been attributed to short-lived abdominal pain, distension and irritable bowel syndrome-like symptoms [10, 11].

### Redundant colon

In contemporary practice, endoscopists, radiologist and surgeons alike frequently note the presence of a ‘redundant colon’ [12–29]. Hanson et al. (2007) defined redundant colon as an elongated or tortuous colon or in the presence of two or more acute flexures [14]. Raahave et al. (2009) recorded colonic redundancy as a sigmoid loop rising above the iliac crests, transverse colon below iliac crests, supernumerary loops of left and right colonic flexures or a combination of these. This study also described increasing symptoms and colonic transit time with increasing numbers of redundant loops of bowel [15].

The term describes the presence of a chronically distended, elongated and tortuous colon, with no obvious cause [30–34]. Validated diagnostic criteria for the diagnosis of a redundant colon remain elusive. Its clinical significance is not readily appreciated or understood. The condition can involve any part or the entire colon, most commonly affecting the sigmoid [30, 32, 33, 35, 36]. Commonly associated symptoms include constipation and gas distress [30, 35–37]. The role of surgical management is debatable, except in instances of acute complications. Its presence is loosely associated with colonic volvulus [36, 38, 39]. Whether it consists of a heterogenous group of conditions or is due to a single cause is unknown [40]. Varying enteric neurochemical and histological findings have been suggested [41–51]. Publications on redundant colon are largely low-level evidence case reports, case series and cohort studies.

While colonoscopy is not a validated tool to diagnose the presence of colonic redundancy, endoscopists often identify the condition in the presence of tortuosity, flaccidity and difficulty maintaining insufflation [14, 35, 37].

### The correlation between diverticulosis and redundant colon

Two historical papers, Ewing (1975) and Goulston (1976), suggested a low incidence of diverticulosis with redundant colon [31, 31, 52]. A common belief also exists among many gastroenterologists and general surgeons that diverticulosis and redundant colon are two conditions rarely seen together. However, no further attempts have been made to substantiate this observation.

This study set out to assess if an inverse relationship exists between diverticulosis and redundant colon. If such a correlation is demonstrated, it may indicate that mechanisms exist leading to the development of one condition and not the other.

## Methods

The James Cook University Human Research Ethics Committee approved this study (approval number H5759) and it was performed in accordance with the recommendation of the Declaration of Helsinki (Edinburgh revision, 2010). Individual consent was obtained from all participants.

### Study design

This was a prospective study performed over 11 months of patients undergoing colonoscopy with participating endoscopists at the Cairns Private Hospital and Cairns Day Surgery, located in Cairns, Australia. Three endoscopists participated in this project—two general surgeons and one gastroenterologist. Each endoscopist had a minimum of 10-year experience.

### Eligibility criteria

Patients undergoing colonoscopy with a participating endoscopist between July 2014 and June 2015, greater than 15 years old, were invited to participate. Those with incomplete data or with a history of colectomy or colostomy were excluded.

### Data measurement and bias

Patients were invited to participate by mail. The presence of redundant colon was recorded as either present or absent. The diagnosis of redundant colon was based upon a subjective finding by the endoscopist of an excessively tortuous, elongated, flaccid or difficult to insufflate colon.

### Statistical analysis

Multivariate binary logistic regression was performed with redundant colon as the dependent variable and gender, age in decades and the presence of diverticulosis as independent variables. Nagelkerke’s *R* ^2^ was calculated to evaluate the goodness of fit to the logistic regression model. To internally validate the multivariate model, we constructed a receiver operator curve (ROC) estimating the area under the curve with 95% confidence interval. Data analysis was performed in SPSS version 22.0 (IBM Corp, Armonk, NY).

## Results

**Fig. 1:**
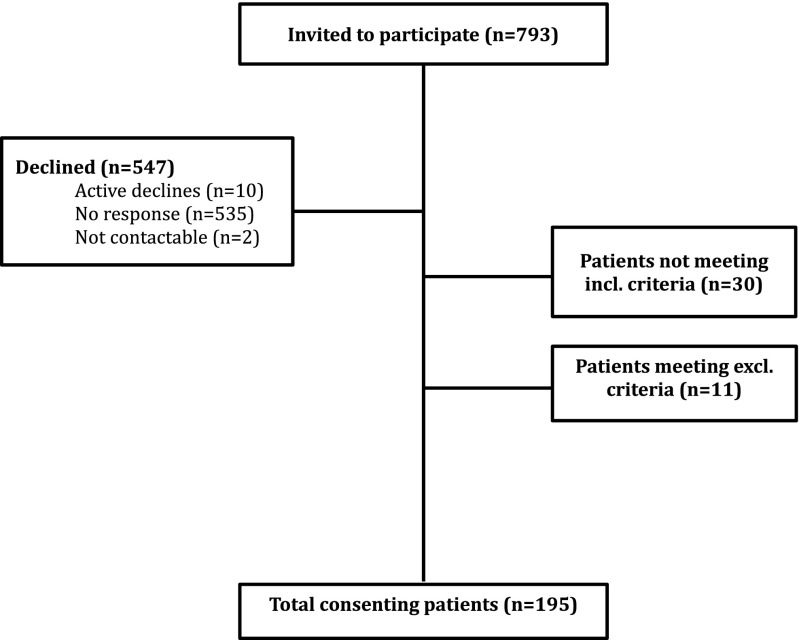
Outcomes of patient recruitment

Of 793 patients undergoing colonoscopy, 195 were included in final data analysis (Fig. 1).

Diverticulosis was diagnosed in 113 (58%) of patients. Redundant colon was diagnosed in 31 (16%) patients. The probability for redundant colon was increased by female gender with odds ratio (OR) 8.4 (95% CI 2.7–26, *p* = 0.00020) and age in decades with OR 1.7 (95% CI 1.1–2.6, *p* = 0.017). The probability for redundant colon was strongly reduced by the presence of diverticulosis with an OR of 0.12 (95% CI 0.42–0.32, *p* = 0.000039). The Nagelkerke *R* ^2^ for the multivariate model was 0.29 and area under the curve at ROC analysis was 0.81 (95% CI 0.73–0.90 *p*–value 3.1 × 10^−8^).

## Discussion

This study verifies the suggestion by Ewing (1975) and Goulston (1976) that the redundant colon and diverticulosis rarely occur concurrently. Based on this study, one can predict the likelihood of a redundant colon occurring in an elderly female without diverticulosis is very high. Comparatively, identifying colonic redundancy in a male with diverticulosis is unlikely. This strong inverse correlation suggests that mechanisms involved with either redundant colon or diverticulosis may protect or impede the development of the other condition. Furthermore, this may provide clues to the pathogenesis.

### Methodological discussion

Patient recruitment for this project was difficult from the outset and throughout. Patient participation required active patient completion of a written consent form that was mailed to them. Unfortunately, in-hospital recruitment could not be performed due to both staffing and ethical restraints. A 30% response rate was obtained. Despite this lower response rate, this study recruited sufficient patients to meet sample size calculations and produce a statistically significant finding.

A validated diagnosis for redundant colon does not yet exist. Colonoscopy is an unvalidated, dichotomous and subjective method to diagnose redundant colon. Endoscopists describe the finding based on their own clinical discretion. Another pitfall of colonoscopy-based diagnosis of redundant colon is the inability to quantify the findings into mild, moderate or severe. Such diagnoses require modalities with physically measurable outcomes—such as radiological imaging or at laparotomy. Some radiological methods such as CT colonography allow for standardized, quantitative diagnoses, which are reproducible between clinicians.

## Conclusion

This study found a statistically significant inverse correlation between the presence of colonic redundancy and diverticulosis during colonoscopy. This finding is in keeping with the suggestions of Ewing (1975) and possibly Goulston (1976) that the two conditions are rarely observed occurring in a single patient. Whether the pathophysiology of colonic redundancy is ‘protective’ against the development of diverticulosis or likewise, it cannot be extrapolated from this study. Further studies unveiling the underlying mechanism for this inverse correlation may also shed light on the pathogenesis of diverticulosis.
